# Guidelines for randomized clinical trial protocol content: a systematic review

**DOI:** 10.1186/2046-4053-1-43

**Published:** 2012-09-24

**Authors:** Jennifer M Tetzlaff, An-Wen Chan, Jessica Kitchen, Margaret Sampson, Andrea C Tricco, David Moher

**Affiliations:** 1 Ottawa Methods Centre, Ottawa Hospital Research Institute, Smyth Road, Ottawa, Ontario, K1H 8L6, Canada; 2 Department of Medicine, Women’s College Research Institute, University of Toronto, Bay Street, Toronto, Ontario, M5G 1N8, Canada; 3 Library Services, Children's Hospital of Eastern Ontario, Smyth Road, Ottawa, Ontario, K1H 8L1, Canada; 4 Li Ka Shing Knowledge Institute of St. Michael’s Hospital, Bond Street, Toronto, Ontario, M5B 1W8, Canada

**Keywords:** Randomized controlled trials, Systematic review, Protocols, Clinical trials, Reporting guideline, SPIRIT initiative

## Abstract

**Background:**

All randomized clinical trials (RCTs) require a protocol; however, numerous studies have highlighted protocol deficiencies. Reporting guidelines may improve the content of research reports and, if developed using robust methods, may increase the utility of reports to stakeholders. The objective of this study was to systematically identify and review RCT protocol guidelines, to assess their characteristics and methods of development, and to compare recommendations.

**Methods:**

We conducted a systematic review of indexed literature (MEDLINE, EMBASE and the Cochrane Methodology Register from inception to September 2010; reference lists; related article features; forward citation searching) and a targeted search of supplementary sources, including a survey of major trial funding agencies in six countries. Records were eligible if they described a content guideline in English or French relevant to RCT protocols. Guidelines were excluded if they specified content for protocols for trials of specific procedures or conditions or were intended to assess trial quality. We extracted guideline characteristics and methods. Content was mapped for a subset of guidelines that described development methods or had institutional endorsement.

**Results:**

Forty guidelines published in journals, books and institutional reports were included in the review; seven were specific to RCT protocols. Only eight (20%) described development methods which included informal consensus methods, pilot testing and formal validation; no guideline described all of these methods. No guideline described formal consensus methods or a systematic retrieval of empirical evidence to inform its development. The guidelines included a median of 23 concepts per guideline (interquartile range (IQR) = 14 to 34; range = 7 to 109). Among the subset of guidelines (n = 23) for which content was mapped, approximately 380 concepts were explicitly addressed (median concepts per guideline IQR = 31 (24,80); range = 16 to 150); most concepts were addressed in a minority of guidelines.

**Conclusions:**

Existing guidelines for RCT protocol content varied substantially in their recommendations. Few reports described the methods of guideline development, limiting comparisons of guideline validity. Given the importance of protocols to diverse stakeholders, we believe a systematically developed, evidence-informed guideline for clinical trial protocols is needed.

## Background

All randomized clinical trials (RCTs) require a protocol describing the rationale, methods, proposed analysis plan and organizational/administrative details from trial inception to reporting of results. Throughout a trial, diverse groups use the trial protocol, including investigators, participants and personnel, funding agencies, research ethics committees/institutional review boards (REC/IRB), journal editors and systematic reviewers. Transparent and clearly written protocols are important to guide trial conduct. They enable thorough assessment of adherence to scientific and ethical standards prior to trial inception [1-6] and monitoring of changes made throughout a trial that could bias interim or final trial results [5]. Some journals now require the submission of protocols with trial manuscripts, which are then included in the peer review process [7-10].

Unfortunately, a high proportion of trial protocols do not adequately describe important methodological details, decreasing their utility for trial implementation and critical appraisal of trials. For example, protocols often fail to designate primary outcomes [11] or detail allocation concealment [12], sample size calculations [13] and sponsor and investigator roles in trial conduct [14], all of which have been associated with biased trial results and conclusions. Additionally, comparisons of trial protocols with corresponding journal publications have consistently shown important, unacknowledged discrepancies, including discrepancies in primary outcomes [5] and statistical methods [13,15]. With recent calls for greater access to trial protocols [16,17] and trial registration [18], the content of trial protocols is receiving increased attention.

Reporting guidelines have been developed to improve the transparency of other research documents such as reports of research findings for journal publication [19-26]. Indeed, the implementation and endorsement of some of these guidelines, including the CONSORT Statement (*CON*solidated *S*tandards *O*f *R*eporting *T*rials) [27] for reports of RCT findings, have been empirically shown to improve report quality [28-30]. However, development methods of reporting guidelines vary, potentially impacting their utility to various stakeholders [20]. Some groups advocate that reporting guidelines should be developed using rigorous, systematic and transparent methodology and recommendations for reporting guideline development have recently been proposed [31].

Guidelines for clinical trial protocol content are available from varied sources, such as textbooks, funding applications and institutional guidelines. However, to our knowledge, their characteristics and methods of development have not been reviewed. In this paper we report a systematic review with the following objectives: 1) to identify reporting guidelines relevant to RCT protocols; 2) to examine their characteristics and development methods; and 3) to review their content.

## Methods

The systematic review protocol was developed with input from trial and systematic review methodologists (See Appendix A in Additional file 1). This report describes the results of an updated review (original completed 2007; JT, AWC, ACT, DM; unpublished).

### Eligibility criteria

Documents were eligible if they included a guideline that explicitly itemized content that should be included in protocols for human RCTs and that recommended methodological details beyond a generic heading for ‘Methods’; the term ‘proposal’ was added to the original eligibility criteria as it is sometimes used to refer to the protocol. Documents describing only common or typical protocol content (without recommending content) were excluded; when intent was unclear, the report was included. Tools were excluded if they recommended content specific to a narrow health care research area (for example, disease stage based on a specific classification system) as we intended to focus on guidelines that could be generalized to other research topics; to guide specific protocol aspects such as quality of life assessment; or to assess clinical trial quality. For practical reasons, guidelines were limited to those available in English or French. Both published and unpublished guidelines were eligible.

### Information sources

Relevant guidelines were identified via two methods: 1) systematic review of indexed literature and 2) targeted search of major RCT funding agencies.

#### Systematic review of the literature

Searches were conducted in MEDLINE including in-process and other non-indexed citations (1948 to September Week 4 2010, Ovid interface); EMBASE including EMBASE Classic (1947 to 2010 Week 40, Ovid interface); and the Cochrane Methodology Register (*The Cochrane Library 2010, Issue 4*, Wiley interface). An information specialist (MS) developed the search strategies. The MEDLINE search, presented in Appendix B in Additional file 1, was amended for the other electronic databases (available upon request). Overlap in journal coverage between MEDLINE and EMBASE was removed using a Batch Citation Matcher technique [32], and remaining duplicates were removed in Reference Manager (Thomson Reuters Corporation, New York, USA). We also searched reference lists of included studies, the PubMed ‘related articles’ link, SCOPUS to identify reports citing the included studies, major clinical trials registries (clinicaltrials.gov, controlled-trials.com), and the EQUATOR Network (*E*nhancing the *QUA*lity and *T*ransparency *O*f health *R*esearch) database of reporting guidelines [19] for additional relevant guidelines. A sample of books was also reviewed and were identified based on book title through reference lists and via searches on Amazon.com [33], WorldCat.org [34], and local library portals using the search terms ‘protocols’ or ‘clinical trials’.

#### Targeted searching of RCT funding agencies

We reviewed a sample of guidelines from major clinical trial funding agencies as we expected some key guidelines would not be readily identifiable through electronic database searching. A short questionnaire was circulated by email to a convenience sample of six key informants who were nominated by the research team, each representing one of six countries previously identified as the top ‘health-related publication producers’ [35]: United States, United Kingdom, Japan, Germany, France and Canada. Informants provided up to two nominations from each of the following sectors within their country: major public (for example, governmental), non-governmental, non-profit (for example, charitable), and for-profit (for example, pharmaceutical industry) clinical trial funding organizations. We reviewed the funding agencies’ websites and contacted the agencies/companies by email to identify relevant guidelines.

### Study selection and data extraction

Two reviewers independently screened titles and abstracts followed by potentially relevant full text articles using pre-defined eligibility criteria and pilot-tested forms. Disagreements were resolved by consensus or by the involvement of a third reviewer.

The following data were extracted from the included studies: report characteristics (publication year/version, source); guideline characteristics (format, intended scope, funding, and endorsement); contributors (number, country of residence and expertise of contributors); guideline development process (including use of consensus methods or evidence to inform content, pilot and validity testing, time-frame and dissemination methods); and guideline content (number and content of recommended items).

Two reviewers independently extracted data from a 25% random sample of studies using a standardized pilot-tested form (JT and JK). The remaining characteristics were then extracted by one reviewer, with key data (methods, search for evidence and number of guideline items) extracted in duplicate independently by a second reviewer. Where multiple reports of a guide or associated website were identified (that is, companion reports), all known associated sources were consulted and the most updated or complete version was treated as the primary source.

#### Guideline content

We itemized guideline content to compare recommendations across guidelines. This analysis was limited to evidence-informed guidelines, those with explicitly described methodology and those with either explicit or probable endorsement of the guideline by a recognized institution or organization. This subset was chosen to select guidelines that were potentially more rigorously developed (that is, those with methodology beyond the consensus of a few authors’ opinions) or more widely acknowledged. To aid in this comparison, we referred to the 2005 version (PR spreadsheet of elements V2.0*;* David Gemzik, personal communication) of Clinical Data Interchange Standards Consortium’s (CDISC) Protocol Representation Model [36], which aims to comprehensively list potential protocol concepts (to support the interchange of protocol information). Guideline content was mapped, where possible, to one of 264 concepts included in this model. Where no suitable concept existed or where the concept had a different level of granularity than the CDISC concepts, a new category was created. Content mapping was conducted by one reviewer (JT) and verified in full by a second reviewer (JK).

### Synthesis of results

Data were summarized using descriptive measures. The following pre-specified sub-groups were compared descriptively: guidelines limited to RCTs *versus* those with a broader scope; guidelines with *versus* those without explicit development methods or cited evidence; and guidelines with *versus* those without described funding sources. A sensitivity analysis compared guidelines explicitly intended for ‘protocols’ *versus* those for ‘proposals’. Due to the nature of the review, no formal reporting bias assessments were conducted.

## Results

**Figure 1:**
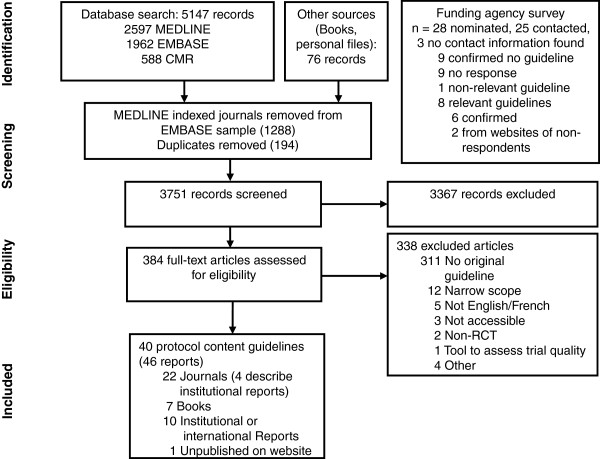
Flow of information through the systematic review.

Electronic searches yielded a total of 5,147 records (Figure 1), and 76 records were identified from other methods. Ten guidelines were identified from nominated funding agencies or their websites, eight of which were relevant; nine agencies confirmed no relevant guideline and no response was received (and no guideline located) for seven agencies (see Appendix C in Additional file 1 for list of agencies). After screening titles and abstracts, 384 full-text documents were reviewed and 46 were included in this review. Six guidelines were presented in two separate reports [37-48], leaving 40 unique guidelines for data extraction [38,40,42,44,46,48-82] (Table 1).

**Table 1:** Guidelines included in this review (primary source)

**Guideline**	**Reference**
*Institutional or named guidelines*	
A method for Rapid, Objective and Structured Evaluation (ROSE) of protocol of a randomised clinical trial (2005).	[56]
A Standard for the Scientific and Ethical Review of Trials (ASSERT) statement (2007).	[71]
Canadian Institutes of Health Research (2010 September) Funding opportunity details.	[75]
Centers for Disease Control and Prevention (2006) Developing a protocol: a guide for CDC investigators.	[69]
Chaput de Saintonge (1977) - produced by past and present members of Clinical Trials Unit, Department of Pharmacology and Therapeutics, London Hospital Medical College.	[55]
Clinical trial protocol templates at the National Institute of Allergy and Infectious Diseases (2009).	[59]
Code of Federal Regulations (2002): U.S. Food and Drugs. 21CFR312.23.	[82]
Code of practice for the clinical assessment of licensed medicinal products in general practice. (1983)	[63]
Guidelines for the preparation of E.R.T.C. cancer clinical trial protocols (1980).	[53]
International Conference on Harmonisation (1998). Harmonised Tripartite Guideline: Guideline for Good Clinical Practice E6 (ICH E6).	[70]
International Ethical Guidelines for Biomedical Research Involving Human Subjects (2002). Council for International Organizations of Medical Sciences (CIOMS) in collaboration with the World Health Organization (WHO)	[42]
Merck investigator studies program protocol template (2008). Requirements for submitting a full proposal.	[74]
National Health Service Department of Health [UK] (2011). Clinical trials toolkit - Based on ‘Detailed guidelines on the principles of good clinical practice in the conduct in the European Union of clinical trials on medicinal products for human use Ver 5.1’	[43,44]
Pfizer Investigator-Initiated Research Program (2010). Investigator-initiated research & requests for pure substance (CTP).	[75]
Programm klinische studien leitfaden für die antragstellung (2010). Deutsche Forschungsgemeinschaft.	[81]
Schneidermann (1961) - Prepared for Cancer Chemotherapy National Service Center of the National Cancer Institute	[66]
The Trial Protocol Tool: The PRACTIHC software tool that supports the writing of protocols for pragmatic randomized controlled trials. (2006)	[46]
U.S. Department of Health and Human Services (2006). Public Health Service Grant Application (PHS 398).	[73]
Warren (1978) - produced by the South-east Thames Regional Health Authority's regional research committee (for which author was chairman)	[54]
Wellcome Trust (2011). Funding for clinical trials. Requirements for applicants.	[80]
Working group of the Commission on Dental Materials, Instruments, Equipment and Therapeutics (1977). Recommended outline for a research protocol.	[40]
*Book chapters*	[38,48,57,58,77-80]
*Other*	[49-52,60-62,64,65,67,68,79]

### Guideline characteristics and methods of development

We present the general characteristics of the guidelines in Table 2. The majority of guidelines were published as journal articles (n = 22; 55%); most were completed/published from 1991 to present (n = 24; 60%); and most were presented as checklists, tables or bullet lists (n = 27; 68%), some with additional explanatory text. Seven (18%) were specific to RCTs [46,50,51,56,65,71,75]. Fifteen guidelines were disseminated via websites (either specific to the tool or independent sites with links to view the guideline), and five reports described conference or lecture presentations [49,51,59,62,73]. Six (15%) guidelines reported a funding source, all of which were non-profit. Most guidelines (n = 25; 62%) had no clear institutional endorsement. Guidelines contained a median of 23 recommended items (IQR = 14, 34; range = 7 to 109).

**Table 2:** General characteristics of included guidelines (N = 40)

**General characteristic**	**n (%)**
** Type of document**	
Journal article	22 (55)
Book	7 (16)
Institutional or international agency guideline not presented in one of the above	10 (25)
Other (1 website)	1 (3)
** Date of publication/version**	
Earlier than 1971	1 (3)
1971-1980	5 (13)
1981-1990	7 (18)
1991-2000	6 (15)
2001-2010	18 (45)
Not stated/unclear	3 (8)
** Format**	
Text (for example, headings)	11 (28)
Checklist/Table/Bullet list	16 (40)
Checklist/Table/Bullet list and additional text	11 (28)
Template	2 (5)
** Scope**	
RCT only	7 (18)
More than RCTs	29 (73)
Unclear/not stated	4 (10)
** Guidance version**	
New guidance	31 (78)
Building on existing guidance	5 (13)
Update of previous guidance	4 (10)
** Funding**	
Yes - non-profit	6 (15)
Not reported/Unclear	33 (83)
Reported no funding	0 (0)
** Endorsement (explicit or probable)**	15 (38)
** Number of items** - median (IQR); range	23 (14, 34); 7 to 109
Number of explicit concepts addressed* - median (IQR); range	31 (24, 80); 16 to 150

*from N = 23 guidelines with description of development methods, use of evidence to support recommendations, or institutional endorsement (explicit or probable).

In Table 3, we report aggregate results of guideline development methodology; details for each guideline are included in Appendix D in Additional file 1.

**Table 3:** Overview of guideline development methods (N = 40)

**Characteristic**	**N (%)**
** Methods described (any)**	
Yes	8 (20)
None reported	19 (48)
Unclear*	13 (33)
** Consensus process**	6 (15)
Formal (for example, Delphi)	0 (0)
Informal consensus and/or consensus meeting(s)	4 (10)
** Pilot testing**	2 (5)
** Validation**	
Formal validation	1 (3)
Informal validation - shared with a broader circle of experts (for example, for face validity)	5 (13)
Future plans to validate	1 (3)
** Search for existing guidance/evidence to inform guideline recommendations**	
Yes	2 (5)
Search for previous guidelines	2 (5)
*Systematic*	0 (0)
Search for empirical evidence	1 (3)
*Systematic*	0 (0)
None reported	34 (85)
At least one empirical study referenced to support at least one guideline item - methods of searching not reported	3 (8)
Unclear	1 (3)
** Number of authors/stated contributors** - median (IQR); range	2 (1, 3); 1 to 88
Not stated	11 (28)
** Time frame for guideline development (years) [n = 4 guidelines]** - median (IQR); range	4 (2, 4); 1 to 5

*If no methods were described, institutional guidelines were inferred to have some form of consensus and were labeled as unclear.

Eleven reports (28%) did not identify the contributors to their development. Of those that did, the majority (n = 24/29, 83%) listed at most 3 contributors (median (IQR) = 2 (1,3)) and most included contributors from a single country (n = 24/29; 83%); exceptions are noted in Appendix D in Additional file 1. Contributors’ areas of expertise were clearly reported for only 8 (20%) guidelines while other reports (for example, [43]) listed names and affiliations of all contributors without explicitly stating their areas of expertise. Contributors’ stated areas of expertise included clinical researchers/clinicians (five guidelines), methodologists/statisticians (two guidelines) and bioethicists, trial managers and information technology personnel (one guideline each).

Only eight reports (20%) described any development methods (Table 3 and Appendix D in Additional file 1): six journal articles [46,54-56,59,63] (two of which were specific to RCT protocols [46,56]), and two international reports [42,70]. Four of these guidelines describe more detailed and comprehensive methodology: the Council for International Organizations of Medical Sciences (CIOMS) International Ethical Guidelines for Biomedical Research Involving Human Subjects [42], the International Conference on Harmonization Tripartite Guideline for Good Clinical Practice E6 (ICH E6) [70], the PRACTIHC tool (Pragmatic Randomized Controlled Trials in HealthCare) [46] and templates developed for the United States National Institute of Allergy and Infectious Diseases [59] (See Appendix D in Additional file 1).

Stated development methods included informal consensus procedures (including consensus meetings) (n = 4; 10%) [42,46,59,70]; pilot testing (n = 2; 5%) [46,59]; soliciting input from a broader stakeholder group (for example, public/experts) prior to dissemination (n = 5; 13%) [42,46,54,59,70] and formal tool validation (n = 1; 3%) [56]. No report stated all of these methods. Two reports included a prospective request for public feedback after dissemination [55,56] and one stated future plans for formal tool validation [59]. No report indicated the use of a formal consensus process (for example, Delphi consensus, Nominal Group Technique) for guideline development.

Additionally, no report described a systematic search for existing guidelines or empirical evidence to inform guideline content. One reported searching personal files [59] for previous guidelines and another reported a non-systematic search of the Internet and reference lists, and contacting experts to identify previous guidelines and evidence [46]. Three reports cited empirical evidence for some of the items [53,58,71] without describing methods for identifying this evidence.

### Guideline content

We extracted content from a subset of 23 guidelines. The recommended content varied substantially between the guidelines (Table 4). Over 380 concepts were recommended (median (IQR) = 31 (24,80) concepts per guideline; range = 16 to 150), over half of which were each recommended in only one guideline (including both distinct concepts such as *conflicts of interest* and sub-concepts of existing headings such as *rationale for choosing specific outcomes*). We present the most commonly recommended concepts in Table 4.

**Table 4:** Common concepts requested in the 23 protocol guidelines with explicit methodology or institutional adoption (numbers in parentheses represent number of guidelines)*

**Concepts in >75% guidelines**	
*General*	*Statistical analysis*
Rationale/purpose (20)	Statistical/analysis methods (general) (21)
Objectives (general) (18)	
**Concepts in 51-75% of guidelines**	
*General*	*Recruitment and eligibility (continued)*
Trial sites/institutions/ location (12)	Sample size + sample size/power calculation (17)
Background (general) (14)	*Treatments and allocation*
*Design*	Treatment/interventions (general) (14)
Study design type/description (17)	*Assessments*
*Recruitment and eligibility*	Outcomes/ endpoints (list) (15)
Eligibility criteria (16)	
**Concepts in 26-50% of guidelines**	
*General*	*Statistical analysis*
Protocol/study title (9)	Interim analysis description/general methods (7)
Principal investigator - name and address (9)	Trial termination criteria/stopping rules (6)
Protocol summary (6)	*Safety and monitoring*
Prior research (literature review) (10)	Assessment of safety - general (7)
Summary of known potential risks/benefits (6)	Safety monitoring/procedures for unscheduled events (9)
*Design*	SAE reporting procedures (to sponsor and regulatory
Statistical hypotheses (8)	authorities) (8)
Study schematic/flow-chart (for example, periods, duration) (6)	Data monitoring committee (role, composition, independence) (7)
Source population/recruitment source (7)	Data management/record keeping (general) (9)
*Recruitment and eligibility*	Methods to ensure data quality/integrity (for example, monitoring, validation) (7)
Recruitment methods/subject selection (11)	*Ethical considerations*
Information for patients and consent/methods (8)	Ethics (general heading) (6)
Eligible concomitant therapies (6)	IRB review/approval/responsible IRBs (7)
Subject drop-out criteria (7)	*Dissemination*
Justification for sample size calculation (6)	Publication policy (general) (6)
*Treatments and allocation*	Disseminating results/publication plan (general) (7)
Description of treatment (10) or comparators (8)	*Other*
Treatment duration (8)	Trial management (general - personnel and administration) (9)
Allocation/randomization methods (general) (10)	Budget (6)
Degree of blind (for example, double, who)/blinding methods (8)	References/cited literature (10)
*Assessments*	Appendices (general) (6)
General (variables and data collection) (10)	Copy of all questionnaires and data forms (8)
Primary endpoint(s)(7), Secondary endpoint(s) (6) (list)	
Methods of assessment/required tests (11)	
Timing of outcome assessment (7)	
Follow-up procedures (7)	
**Concepts in ≤ 25% of guidelines** (n = 333; subset provided below)	
*General*	*Treatments and allocation (continued)*
Site investigators/collaborators - names and addresses (4)	Reasons for degree of blinding (3)
Sponsor - name and address (5)	*Assessments*
Registration plans/registration number (4)	Assessment of compliance (5)
Protocol identifying number (5)	Validity/reliability of collection/measurements (1)
Funding source (3)	*Statistical analysis*
Rationale with reference to a systematic review (3)	Description of planned subgroup analyses (2)
*Recruitment and eligibility*	*Other*
Target population (5)	Participant security/confidentiality (5)
Justification for special (for example, vulnerable) pop. (5)	Study timetable (calendar (date) of events) (5)
Specific eligibility criteria - for example, health status (2), co-enrolment in trials (2)	Target duration for trial as a whole (5)
*Treatments and allocation*	Approximate time to complete enrolment (5)
Treatment dosage (5), route of admin. (5), justification (5)	Dummy tables (3)
Form and location of treatment code (5)	Curriculum vitae of investigators (5)
Allocation concealment (3), Implementation of randomization (1)	Incentive to investigators/staff (1), participants (2)
	Conflict of interest (1)
	Consumer involvement (who and roles) (1)

#### Subgroup comparisons

Few differences were noted between pre-specified subgroups by scope, development methods, and funding source. The number of guidelines in each subgroup was small, thus limiting the ability to make definitive conclusions. In Table 5 we present the most notable differences between the subgroups. No differences were found between guidelines intended for ‘protocols’ *versus* those for ‘proposals’.

**Table 5:** Major differences between pre-defined subgroups*

**Methods**	**Recommendations**
*Guidelines specific to RCTs versus those with a broader scope (RCT + other study designs)*	
No differences	RCT only guidelines are more likely to recommend including:
	· a systematic review as part of study background
	· reason for degree of blinding
	· methods to generate allocation sequence
	· methods to implement randomization (who will generate sequence, who will enroll participants, who will allocate participants)
*Guidelines with explicitly described methods of development versus those without*	
Those with explicit methods are more likely to:	Those with explicit methods are more likely to recommend including:
· describe multiple dissemination methods	· Procedures to break the blind
	· Specific issues of consent with respect to vulnerable populations (for example, children, non-literate populations)
	· Dosing frequency
	· Duration of subject participation
*Guidelines with explicit sources of funding versus those without*	
Funded guidelines are more likely to:	Guidelines with funding are more likely to recommend including:
· Have explicitly described methods (including informal consensus methods, searching for evidence, informal tool validation)	· Procedures to break the blind
	· Specific issues of consent with respect to vulnerable populations (for example, children, non-literate populations)
· More likely to describe multiple dissemination methods	
	· Dosing frequency
· Have multiple contributors (Median [IQR] = 9 [3,15]*versus* 1 [1,3])	· Site representative/investigator
	· Time and event schedules table

*Subgroups were compared descriptively; as sample sizes were small only major differences are noted. IQR: Inter-quartile range; RCT: randomized clinical trial.

## Discussion

Our review identified numerous guidelines aiming to inform the content of clinical trial protocols. However, recommended concepts varied substantially across guidelines and the vast majority of guidelines did not describe their methods of development. When described, most included informal methods with limited stakeholder involvement and limited use of evidence to inform their recommendations. Similar findings have been reported elsewhere [20,][83].

Very few concepts were recommended consistently across guidelines, including several whose importance is supported by empirical evidence. For example, only half of the more recent guidelines [59,70,71,74,75,80,81] included an item recommending that primary outcomes be stated, despite preceding research showing biased modifications of primary outcomes throughout trials [3,5,11]. Similarly, only three [6,66,71] explicitly requested information regarding allocation concealment, the absence of which has been associated with inflated trial effect sizes [84-86], although many requested general allocation methods. Conflicts of interest and roles of the sponsor in the trial were explicitly recommended in only one guideline [81], despite being required by the Declaration of Helsinki [87] and despite research showing that trials with financial competing interests report positive results more often than other trials [6,88,89]. Only three [71,75,81] guidelines explicitly recommended including or citing a systematic review as part of the trial rationale despite the problems associated with non-systematic literature searches [90,91]. Finally, only 4 [46,71,72,81] of 15 guidelines published after the introduction of trial registration requirements in 2005 [18] specifically requested registration information. No guideline recommended all of these important concepts.

The reasons for the variation and omissions are unclear. Few of the guideline reports in our sample described their development methods, preventing assessment of the validity of the recommendations. If not properly developed, guidelines could potentially ultimately be of limited use and may not improve the reporting of elements that are important to key users of protocols. Of the eight guidelines that did detail methodology, four seem relatively comprehensive [42,46,59,70]. Although these four shared many common elements, considerable variation in recommended content was also present.

For a guideline to be widely acceptable, we believe it should be developed using robust methodology that engages key stakeholders during development and is guided by empirical evidence, where possible. In addition, the methodology should be clearly reported and accessible to enable understanding of the process and assessment of its validity. Recommendations for reporting guideline development have recently been proposed [31] and include a series of steps akin to those recommended for clinical practice guideline development [92]: involvement of multidisciplinary expert panel for a formal consensus process (for example, Delphi consensus) and consensus meeting(s), literature reviews to identify key evidence, pilot testing, active dissemination and impact evaluation. Recent research conducted by the EQUATOR group on the development of health care reporting guidelines [20,83] suggests that such extensive methods are rarely employed. This is congruent with our current findings.

This review has some limitations. Although comprehensive in searching indexed periodicals, our review was not exhaustive in the search for institutional guidelines or books. However, our main findings would not likely substantively change with the inclusion of guidelines from these sources, as most guidelines available outside of journal articles did not describe development methods. Our results are also based on the methodology stated in included reports; we did not contact authors for additional information. Finally, the process of mapping and comparing concepts across guidelines was challenging due to the varied terminology used and the many sub-concepts of general headings that were recommended. To decrease bias we employed a systematic method and a second reviewer verified the process.

Our systematic review highlights some potential limitations of existing clinical trial protocol content guidelines. Given the evidence of protocol deficiencies [11-14] and the importance of trial protocols to diverse stakeholders we believe there is a need for standard guidance that is developed using rigorous methods, broad consultation with key stakeholders and is based on empirical evidence, where possible. Development of reporting guidelines requires substantial resources and time [31], and the conduct of this review is an important inaugural step to justify undertaking such an initiative. Since the initial version of this review, an international collaboration known as the SPIRIT Initiative (*S*tandard *P*rotocol *I*tems: *R*ecommendations for *I*nterventional *T*rials) has convened to produce such guidance by systematically developing recommendations for minimum content of clinical trial protocols [93]. The primary aim of SPIRIT is to improve the content and utility of clinical trial protocols.

## Conclusions

This review identified many guidelines for clinical trial protocols; the recommendations provided by these guidelines varied substantially and potentially important concepts were often not recommended. Most guidelines did not describe their methods of development and none of the reports described replicable methods of development including formal consensus of key stakeholders or a thorough search for relevant empirical evidence. Given the importance of trial protocols to diverse stakeholders and evidence of protocol deficiencies, we believe that development of a guideline meeting such standards is needed.

## Abbreviations

CDISC: Clinical Data Interchange Standards Consortium; CIOMS: Council for International Organizations of Medical Sciences; CONSORT: CONsolidated Standards Of Reporting Trials; EQUATOR: Enhancing the QUAlity and Tranparency Of Health Research; ICH E6: International Conference on Harmonization Tripartite Guideline for Good Clinical Practice E6; IQR: interquartile range; IRB: Institutional Review Board; PRACTIHC: Pragmatic Trials In Health Care; RCT: Randomized Clinical Trial; REC: Research Ethics Committee; SAE: Serious adverse event; SPIRIT: Standard Protocol Items, Recommendations for Interventional Trials.

## Competing interests

The authors have declared that no competing financial interests exist. Three members of the review team (JT, DM and AWC) are involved in the SPIRIT initiative.

## Authors' contributions

JT prepared the protocol with guidance from DM, AWC, ACT, and MS. MS and JT developed the search strategies. JT and JK selected relevant studies and extracted data; ACT participated in screening/extraction for the initial unpublished version of the review. JT carried out the analysis and prepared the manuscript with input from all authors. All authors read and approved the final manuscript.

## Financial disclosure

No direct funding was received for this study. Some authors were personally salaried by their institutions during the period of writing though no specific salary was set aside or given for the writing of this paper. Dr. Moher is supported, in part, by a University (of Ottawa) Research Chair. No funding bodies had any role in the study design, data collection, analysis, decision to publish or preparation of the manuscript.

## Supplementary Material

Additional file 1 **Appendix A.** Systematic review protocol. **Appendix B. **Search strategy for Ovid MEDLINE® (including in-process and other non-indexed citations) 1948 to September Week 4 2010. **Appendix C. **Results of contact with nominated trial funding agencies. **Appendix D.** Details of guideline development methods described in reports (N = 40 guidelines). Click here for file
