# Supplementary material for: Guidelines for randomized clinical trial protocol content: a systematic review
Source: Syst Rev. 2012 Sep 24;1:43. doi: 10.1186/2046-4053-1-43 (PMC3533811; doi:10.1186/2046-4053-1-43)
Supplement: Additional file 1 — Appendix A. Systematic review protocol. Appendix B. Search strategy for Ovid MEDLINE® (including in-process and other non-indexed citations) 1948 to September Week 4 2010. Appendix C. Results of contact with nominated trial funding agencies. Appendix D. Details of guideline development methods described in reports (N = 40 guidelines). [file 2046-4053-1-43-S1.doc]

**Appendix A: Systematic review protocol**

**Please note:** The following document is the protocol to the initial version of the submitted systematic review entitled: “Guidelines for randomized controlled trial protocol content: a systematic review”. The initial version was conducted in 2007.

The methodology for the submitted updated version has been modified to improve retrieval of relevant records, enhance data extraction and improve internal validity of the research.

The following describes the changes to the review methods from the attached protocol – all changes were approved by JMT, AWC, DM and JK prior to commencing this update with the exception of #2 which was approved during screening to increase specificity. Changes to the search strategy were developed and approved by MS.

1. Objective ‘synthesizing the evidence used to inform development of guidelines’ was not included in update
   - Reason: was not considered pertinent to primary goals of the updated review.
2. Eligibility criteria were clarified to attempt to exclude reports limited to ‘typical’ or common protocol content. We also excluded guidelines specific to particular aspects of the research protocol, such as issues related to quality of life assessment**.**
   - Reason: clarification/increasing specificity
3. The electronic search strategies were modified and updated to improve retrieval of relevant records**.**
   - Reason: increasing retrieval and specificity
4. All screening was conducted by two reviewers (rather than by one with a random sample conducted by a second reviewer).
   - Reason: increasing internal validity
5. For primary outcomes, data extraction was conducted in duplicate; a 25% random sample of the remaining outcomes were extracted in duplicate (rather than 10% for all outcomes as previously specified).
   - Reason: increasing internal validity
6. Method of synthesizing concepts included in guidelines made more systematic with the use of a previous version of CDISC’s (Clinical Data Interchange Standards Consortium) Protocol Representation Model (please see final manuscript for references) and by verification by a second reviewer.
   - Reason: increasing internal validity
7. Subgroup analysis not conducted for guidelines in indexed literature vs. those identified by survey of key informants
   - Reason: guidelines overlapped categories
8. Sensitivity analysis added for guidelines intended for ‘protocols’ versus those intended for ‘proposals’ or where intent was unclear
   - Reason: To examine if this change in search strategy and eligibility affected results
9. Data extraction questions were modified as appropriate for clarification and for extraction into spreadsheets.

**A systematic review of reporting guidelines for randomized controlled clinical trial protocols (Protocol)**

**Submitted by: Jennifer Tetzlaff**

**February 16^th^, 2007**

**Submitted to: Drs. Dean Fergusson and David Moher**

**EPI 6188**

**Systematic Review Team**

**Tetzlaff J^1,2^, Tricco A^1,3^, Moher D^1,4^, Chan A-W^5^**

^1^Department of Epidemiology and Community Medicine, University of Ottawa, Ottawa, Canada; ^2^Chalmers Research Group, Children’s Hospital of Eastern Ontario Research Institute; ^3^Institute of Population Health, University of Ottawa, Ottawa, Canada; ^4^Faculty of Medicine, Univerisity of Ottawa, Ottawa, Canada; ^5^University of Toronto, Toronto, Canada.

**Abstract**

This is a protocol for a review and there is no abstract.

The objectives are as follows:

To identify existing guidelines for reporting randomized controlled clinical trial protocols and to summarize development methods and items included in these existing guidelines.

**Background**

Every high quality randomized clinical trial requires a protocol describing the trial rationale, methods, proposed analysis and organizational/administrative details of the trial. Protocols should be a comprehensive account of the proposed trial methodology, from trial inception to publication of the research results. Protocols are essential to promote experimental research that is scientifically and ethically sound and that meets standards for protection of human subjects.

Clinical trial protocols are important for many reasons. They provide investigators with a central document to guide conduct throughout the trial; they provide trial participants or potential participants with a detailed description of trial methodology; they offer ethics committees/institutional review boards (REC/IRBs) a foreknowledge of predefined safeguards to protect participants’ interest; and they serve to inform funding agencies of the planned research and provide a means of accountability for adherence to proposed methods. As such, the reporting of clinical trial protocols is extremely important.

The reports of clinical trial protocols must be clear, detailed and transparent, not only for reasons of practicality as outlined above, but also to serve to protect the trial from sources of bias. Bias may affect a trial throughout the period of its conduct (e.g. recruitment, interim analyses, stopping rules), and at completion (e.g. analysis, publication) (1). For example, the absence of allocation concealment at the time of recruitment, lack of blinding and inappropriate randomization of subjects have been shown to bias estimates of effects in clinical trials (2, 3). Protocol development is an opportune time to locate deficiencies in design to minimize chances of such potentially avoidable biases. Therefore, it is imperative that a report of a clinical trial protocol be clearly written such that its methodology can be accurately assessed by researchers, funding agencies and REC/IRBs.

The explicit description of methods and analyses in protocols prior to trial inception is also important to ensure that biased changes are not made based on the interim or final results. Comparisons of trial protocols with corresponding journal publications have consistently shown important and biased deviations. For example, such surveys have shown that primary outcomes are modified between the protocol and the final report in approximately half of all trials (e.g. 4, 5). In one survey, approximately one-quarter of trialists completely omitted a primary outcome in the final report which was defined in the protocol, and approximately one-fifth of trialists reported a new primary outcome in the final report which was not mentioned in the protocol (4). In addition, outcomes which were reported in final reports were significantly more likely to be statistically significant than those omitted. This type of bias has been termed ‘selective outcome reporting bias’. It should be noted, that while there are often valid reasons for changing trial methods, these changes should be made explicit, should be approved by REC/IRBs and should be acknowledged in trial reports, allowing readers the opportunity to assess the potential for bias.

To attempt to monitor such problems, some journals require that protocols be submitted with trial manuscripts for review during publication consideration (e.g 6, 7). However, even with such initiatives, the absence of complete reporting in clinical trial protocols makes it difficult to compare protocols and final reports. A high proportion of trial protocols have been found to contain unclear descriptions of important methodological details; or lack them all together. For example incomplete reporting has been shown for factors such as primary outcomes (4), allocation concealment (8), power calculations (Chan AW et al, personal communication) and the roles of sponsors and investigators in all aspects of trial conduct (e.g. 9).

Incomplete reporting is in no way specific to clinical trial protocols. Countless studies have provided evidence for such inadequacies in many specialties and study designs (e.g. 10, 11). A number of initiatives have set out to improve the quality of reports using evidence, where possible, to guide recommendations. For example, the well-endorsed revised CONSORT Statement (12), a reporting guideline aimed at increasing the transparency of reports of 2-parallel group randomized controlled trials has prompted the development of extensions for other study designs such as cluster randomized trials (13), and the development of independent initiatives such as for studies of diagnostic accuracy (STARD; 14) and systematic reviews (QUOROM; 15*)*. The initiatives have been mostly well-received, and in the case of CONSORT, have been shown empirically to improve the quality of reporting (16). In addition, at least one clinical trial funding agency previously asked that grantees consider how they have dealt CONSORT items in their applications (17). While this to be commended, there is incomplete overlap between the reporting of clinical trial protocols and trial results. For example, clinical trial protocols often require a detailed account of administrative/trial management issues which will not be generally covered in the report of the results of a clinical trial.

Guidelines also exist to inform the reporting of clinical trial protocols and are available from many sources such as textbooks, funding applications and institutional guidelines It is not clear to what extent their development has utilized an ‘evidence-based approach’ that defines many of the guidelines previously cited. For example, what methods were used to develop these guidelines and what evidence, if any, was used to guide their development? In addition, what is the consistency with which they recommend reporting various components of clinical trial protocols?

In the era of calls for greater access to clinical trial protocols and clinical trial registration (e.g. 18), we believe this is an opportune time to review current recommendations for reporting randomized clinical trial protocols, assess the methods used to develop those guidelines and consider the levels of evidence from which they have been derived.

**Objectives**

The objectives of this systematic review are to:

1. identify reporting guidelines for randomized controlled clinical trial protocols;
2. compare characteristics and methods of development of the guidelines;
3. compare items included in the guidelines; and
4. synthesize evidence used to inform the development of guidelines, if relevant.

**Criteria for considering studies for this review**

**Types of studies**

Studies will be eligible for inclusion in this review if they describe a guideline for reporting randomized controlled clinical trial protocols. A reporting guideline will be defined as an itemized guide explicitly informing the content or major headings suggested for inclusion in a protocol. Guidelines must include, but need not be limited to, criteria for reporting protocols for any type of randomized controlled clinical trial (e.g. parallel-group randomized clinical trials, cross-over trials, non-inferiority trials, cluster randomized trials), limited to research in humans. The rationale for limiting the review to reporting guidelines for protocols of randomized controlled trials are that these trials are likely to provide more dependable information than other sources of evidence on effects of healthcare (3). Guidelines specific to particular aspects of the research protocol, such as issues related to quality of life assessment, will be included.

Studies will be excluded if they are intended solely for the reporting of non-randomized, non-controlled clinical trials or if the reporting guideline focuses on reporting protocols of a narrow area of health care research, such as a specific medical procedure, condition or laboratory test. Tools or checklists intended to assess quality of clinical trial protocols, if relevant, will be excluded as these concepts are not synonymous to the development of the protocol.

There will be no exclusions based on the methodology of guideline development as one of the purposes of this study is to compare the development methods, if reported. For practical reasons, guidelines included in this review will need to be published in English or French. However, guidelines published in other languages will be noted and may be included in future revisions of this work. There will be no limits based on publication status of the reporting guidelines.

**Types of Data**

Types of data to be included in this review will be the details of the development methods of reporting guidelines for clinical trial protocols, if relevant; the items included in the reporting guidelines; and evidence supporting the inclusion of that item, if described.

**Outcomes**

The most important outcome defined *a priori* is the extent to which available guidelines are developed based on evidence. Outcomes will include:

- Intended scope of guideline
- Guideline development methods
- The use of evidence to guide development and type of evidence, if relevant
- Dissemination and uptake of guideline
- Funding for the development of guideline
- Items included in reporting guideline

**Search strategy for identification of studies**

Relevant guidelines will be identified via two methods:

1. Systematic review of the literature; and
2. Survey of key informants

**Systematic review of the literature**

*Electronic Sources*

Electronic searches will be conducted in the following databases with no language restrictions:

- MEDLINE (From 1950 to last day of search [will be reported in final report], Ovid interface);
- EMBASE (1980 to last day of search [will be reported in final report], Ovid interface); and
- The Cochrane Methodology Register (The Cochrane Library 2007, Issue 1, Wiley interface). This register is a bibliography of publications that report on methods used in the conduct of controlled trials. It contains over 9000 references for journal articles, books, and conference proceedings that have been identified from MEDLINE and hand-searching of journals.

Search strategies have been developed by JT in consult with an information specialist (Margaret Sampson, MLIS, Chalmers Research Group). The MEDLINE search strategy (1950 to February Week 1 2007, Ovid interface), including output, is reproduced below. Adjustments will be made to the search when run in EMBASE and the Cochrane Methodology Register to take into account differences in indexing. All records will be downloaded and imported into Reference Manager 11 where duplicate records will be removed. All search strategies will be rerun prior to completion of this review. Due to the high retrieval from the search strategy, in the event that time does not permit, a subset of the reviews below (limited by year) will be reviewed for the content of this course.

**MEDLINE (Ovid interface) search strategy:**

| 1 | exp research design/ | 213596 |
| --- | --- | --- |
| 2 | clinical protocols/st, mt | 2135 |
| 3 | Clinical Trials/st, mt | 7836 |
| 4 | biomedical research/ | 12606 |
| 5 | writing/ | 9037 |
| 6 | Or/1-5 | 240621 |
| 7 | Protocol$.ti,ab. | 123376 |
| 8 | (report$ or guideline$ or checklist$ or recommend$ or standard$ or require$ or instruct$ or guidance$  or Aide memoir$ or writ$).tw. | 2570991 |
| 9 | and/6-8 | 3528 |
| 10 | clinical trial.pt. | 431637 |
| 11 | animal/ not human/ | 3028872 |
| 12 | 9 not (10 or 11) | 1818 |

Results from search run 15/02/2007

*Other sources*

- Citation snowballing: SCOPUS will be searched to track publications citing included studies
- PubMed related articles feature will be utilized for included studies.
- Reporting guidelines cited by major clinical trials registries (e.g. clinicaltrials.gov, controlled-trials.com)
- Reference lists of included studies
- Book chapters (Freeman, Spilker, Meinhert…others?)
- Conference proceedings, if relevant. No relevant conference proceedings have yet been identified.
- Contact with authors to identify additional studies.

**Surveys of key informants**

This portion of the review will attempt to identify protocol guidelines of major national or international agencies which fund clinical trials. We will not attempt to collate an exhaustive list of funding agencies, but rather obtain a purposive sample of funding bodies.
 The search for these guidelines will be via a short survey of six key informants. A convenience sample will be chosen such that informants represent one of six countries which have been previously identified as the top six “health-related publication producers” (19): U.S.A., United Kingdom, Japan, Germany, France and Canada. Informants will be sent a cover letter explaining the current review and a brief survey by email, asking them to list up to three major national and one major international funding agency for clinical trials. The corresponding websites of the funding agencies identified via this survey will be accessed to ascertain the presence of a reporting guideline or reference to a reporting guideline for clinical trial protocols. If none are found, the agencies will be contacted to request this information, if available. This survey will be developed by the review team.

# Methods of the review

The conduct and reporting of this systematic review will follow the guidelines outlined in the Cochrane Handbook (20) and the PRISMA Statement (update of the QUOROM Statement, Moher D. *et al.*, personal communication).

**Identifying relevant studies**

***Literature Search***

One reviewer will perform a broad screen of titles and abstracts identified by the literature search to exclude those records that clearly do not meet the inclusion criteria, with a second reviewer verifying a 5% random sample. Two reviewers will then independently screen the title and abstract of all remaining records in duplicate using the Level 1 Screening Form (Appendix A). Full-text articles will be obtained for potentially relevant studies and those where relevance remains unclear. Full-text articles will be independently screened by two reviewers using the Level 2 Screening Form (Appendix A). All disagreements will be resolved by consensus or, if necessary, by the involvement of a third member of the review team. Reviewers will not be blinded to any characteristics of the reports. One reviewer will search reference lists, book chapters, related articles features, conference proceeding, if relevant, and attempt to contact authors of all included studies to identify additional relevant studies. All additional identified articles will be screened according to Level 2 Screening criteria.

***Funding Agency Guidelines***

Clinical trial funding agencies identified from six key informants will be contacted to establish if a reporting guideline for clinical trial protocols exists for their agency. If so, this will be obtained and screened according to the Level 2 Screening criteria as outlined in Appendix A.

A QUOROM-like flow diagram will be used to report the flow of articles/guidelines through the review process (15).

##### Data extraction

The Data Extraction form will be pilot tested by two reviewers on a separate sample of articles included in a previous review of trial reporting guidelines (Simera I *et al.*, 2007, personal communication). A preliminary version of this form is available in Appendix B. This form may be modified following pilot testing. Data extraction will be conducted by one reviewer, with a second reviewer independently extracting data from a 10% random sample of the included studies. All disagreements will be resolved by consensus or, if necessary, by the involvement by a third member of the review team. Should many disagreements arise in this random sample (e.g. >1% disagreement), all data abstraction will be done in duplicate by two independent reviewers.

The following data will be extracted from the included studies:

- Report characteristics (authors, number of authors, country of corresponding author, year of publication, published/not-published)
- Characteristics of the guideline (format, intended scope)
- Accountability (authors, date/version, contact information)
- Guideline development process (types of methods, number of participants, country of participants, role of participants, time-frame of process)
- Internal/external validity (use of evidence in development process, circulation of document for expert validation within or outside working group)
- Dissemination, uptake and impact
- Funding
- Guideline items (number of items, specific items, evidence to support items, if applicable)

All efforts will be made to ensure no duplicate information is included. Corresponding authors will be contacted by email where contact details are supplied to obtain missing information or to clarify existing information. Basic study characteristics of reporting guidelines for controlled clinical trial protocols specific to a certain disease or condition will also be recorded and will be included in a table of excluded studies.

**Assessment of methodological quality**

Due to the nature of the included studies and lack of appropriate tools, no formal quality assessments will be completed for the included studies. Information will be extracted to assess the external and internal validity of the information in each included study.
Information such as the following will be used in this assessment:

- Methods described for the development of the reporting guideline, if any.
  - Methods will be appraised on the bases of external (e.g. dissemination for peer review to a wide expert audience) and internal (e.g. use of evidence to support the inclusion of items) validity.
- Evidence provided supporting the inclusion of items in the reporting guideline, if any.

No reports will be excluded based on methods for tool development or presence/absence of evidence supporting the inclusion of items in the guideline.

**Data analysis**

The kappa coefficient will be used to measure agreement between reviewers at both levels of screening (21). No measures of effect are relevant for this study. The unit of analysis will be both at the level of the reporting guideline and at the level of each item included in the guidelines.

Items included in each guideline will be synthesized using a table, accounting for distinct items reported in the literature, whether or not evidence was used to support the inclusion of the item in the guideline and, if so, the nature of this evidence (e.g. empirical, expert consensus). An example of such a table is available in Appendix C. This table will be modified to synthesize appropriate items with the identification of new items. Item titles may be modified from original guidelines to account for similar concepts.

*Sub-group analyses*

Separate analyses may be considered based on the following characteristics:

- reporting guidelines specific for reporting the protocols randomized controlled clinical trials and those for reporting protocols of other trial designs in addition to randomized controlled trials,
- reporting guidelines reported in the literature versus those specific for clinical trial funding agencies identified by key informants,
- reporting guidelines with development methods versus those without any described methods, and
- reporting guidelines citing evidence supporting items included in guideline versus those not citing any evidence.

Relevant outcomes considered for subgroup analysis will be dependent on specific analysis but will include characteristics of the report, characteristics of the guideline, methods of dissemination, etc.

Due to the nature of the review, no formal assessment of heterogeneity, publication or other reporting biases will be completed.

*Missing data*

An attempt will be made to contact all corresponding authors to obtain missing data.

*Methods for future updates*

The literature search will be run once at the inception of this study and again close to the completion of the study to ensure the currency of the information.

*Protocol Modifications*

All efforts have been made to define *a priori* the information of importance from the included studies. Any changes required between information stated in this protocol and the systematic review will be distinguished in the final report.

**Potential conflicts of interest**

Three members of the review team (JT, DM, AWC) are involved in an initiative to develop an evidence-based reporting guideline for protocols for 2-group randomized controlled clinical trials.

**Acknowledgments**

We would like to extend our gratitude to Dr. Iveta Simera for sharing with us a recent review of existing reporting guidelines. This review influenced the development of the search strategy, screening criteria and data extraction forms for the current review.

Thank you also to Margaret Sampson, MLIS, who helped refine the MEDLINE search strategy.

While the conduct of this review is not financially supported, the results will be included as part of a project funded by the Canadian Institutes of Health Research, Canadian Agency for Drugs and Technologies in Health and the National Cancer Institute of Canada.

**Sources of Support**

**External sources of support**

No sources of support supplied

**Internal sources of support**

No sources of support supplied

**References**

1. Gluud LL. Bias in Clinical Intervention Research. American Journal of Epidemiology. 2006; **163** (6): 493–501.
2. Schulz KF, Chalmers I, Hayes RJ, Altman DG. Empirical evidence of bias. Dimensions of methodological quality associated with estimates of treatment effects in controlled trials. *Journal of the American Medical Association.* 1995; **273** (5): 408-12.
3. Kunz R, Vist G, Oxman AD. Randomisation to protect against selection bias in healthcare trials. *Cochrane Database of Methodology Reviews* 2002, Issue 4. Art. No.: MR000012. DOI: 10.1002/14651858.MR000012.
4. Chan A-W, Hrobjartsson A, Haahr MT, Gøtzsche PC, Altman DG, Empirical Evidence for Selective Reporting of Outcomes in Randomized Trials: Comparison of Protocols to Published Articles. *JAMA.* 2004; **291** (20): 2457-2465.
5. Chan A-W, Krleza-Jeric K, Schmid I, Altman DG. Outcome reporting bias in randomized trials funded by the Canadian Institutes of Health Research. *CMAJ.* 2004; **171** (7): 735-40.
6. Horton R. Pardonable revisions and protocol reviews. *Lancet*. 1997; **349** (9044):6.
7. British Medical Journal. Article Requirements [http://resources.bmj.com/bmj/authors/article-submission/article-requirements]. 2007 BMJ Publishing Group Ltd. [accessed 15 Feb 2007].
8. Pildal J, Chan A-W, Hrobjartsson A, Forfang E, Altman DG, Gotzsche PC. Comparison of descriptions of allocation concealment in trial protocols and the published reports: cohort study. *British Medical Journal*. 2005; **330** (7499): 1049.
9. Gøtzsche PC, Hróbjartsson A, Johansen HK, Haahr MT, Altman DG, et al. Ghost Authorship in Industry-Initiated Randomised Trials. *PLoS Medicine* 2007; **4**(1): e19 [doi:10.1371/journal.pmed.0040019](http://dx.doi.org/10.1371/journal.pmed.0040019)).
10. Scales CD, Norris RD, Keitz SA, Peterson BL, Preminger CM, Vieweg J, Dahm P. A critical assessement of the quality of reporting of randomized, controlled trials in the urology literature. *Journal of Urology*. 2007; **177** (3): 1090-5.
11. Eldridge SM, Ashby D, Feder GS, Rudnicka AR, Ukoumunne OC. Lessons for cluster randomized trials in the twenty-first century: a systematic review of trials in primary care. *Clinical Trials*. 2004; **1**: 80-90. DOI: 10.1191/1740774504cn006rr
12. Moher D. Schulz KF. Altman DG. The CONSORT statement: revised recommendations for improving the quality of reports of parallel-group randomised trials. *Lancet*. 2001; **357** (9263):1191-4.
13. Campbell MK, Elbourne DR, Altman DG. CONSORT statement: extension to cluster randomised trials. *British Medical Journal.* 2004; **328**: 702-8.
14. Bossuyt PM. Reitsma JB. Bruns DE. Gatsonis CA. Glasziou PP. Irwig LM. Moher D. Rennie D. de Vet HC. Lijmer JG. Standards for Reporting of Diagnostic Accuracy. The STARD statement for reporting studies of diagnostic accuracy: explanation and elaboration. *Clinical Chemistry*. 2003; **49** (1):7-18.
15. Moher D. Cook DJ. Eastwood S. Olkin I. Rennie D. Stroup DF. Improving the quality of reports of meta-analyses of randomised controlled trials: the QUOROM statement. Quality of Reporting of Meta-analyses. *Lancet*. 1999. **354** (9193):1896-900.
16. Plint AC. Moher D. Morrison A. Schulz K. Altman DG. Hill C. Gaboury I. Does the CONSORT checklist improve the quality of reports of randomised controlled trials? A systematic review. *Medical Journal of Australia.* 2006; **185** (5):263-7.
17. O'Toole LB. MRC uses checklist similar to CONSORTs. *British Medical Journal.* 1997; **314**:1127.
18. De Angelis C. Drazen JM. Frizelle FA. Haug C. Hoey J. Horton R. Kotzin S. Laine C. Marusic A. Overbeke AJ. Schroeder TV. Sox HC. Van Der Weyden MB. International Committee of Medical Journal Editors. Clinical trial registration: a statement from the International Committee of Medical Journal Editors. *Annals of Internal Medicine.* 2004; **141** (6): 477-8.
19. Paraje G, Sadana R, Karam G. Increasing International Gaps in Health-Related Publications. 2005; **308**: 959-60.
20. Higgins JPT, Green S, editors. Cochrane Handbook for Systematic Reviews of Interventions 4.2.6 [updated September 2006]. In: The Cochrane Library, Issue 4, 2006. Chichester, UK: John Wiley & Sons, Ltd.
21. Landis JR, Koch GG. The Measurement of Observer Agreement for Categorical Data. *Biometrics*. 1997; **33** (1):159-174.

**Contribution of author(s):** JT has prepared this protocol with guidance from DM and AWC and edits from AT. JT will perform all searches. JT and AT will select relevant studies to include, and extract data from included studies. JT will carry out the analysis. JT will prepare and others will comment on the review.

**Contact address:** Ms. Jennifer Tetzlaff

Department of Epidemiology and Community Medicine

University of Ottawa

Ottawa

Ontario

Canada

E-mail: jtetzlaff@cheo.on.ca

Tel: 613-737-7600 x3279

**Appendix A - Screening Forms**

Please note that all screening will be performed using a Reference Manager Database.

**Screening: Level 1**

**Reviewers Initials: _______________**

**REFID: _______________**

1. Does this paper describe a reporting guideline for randomized controlled clinical trial protocols? *Note: The guideline may be relevant to other study designs as well.*

[ ] No (exclude)

[ ] Yes (include) – please answer question 2, if relevant

[ ] Don’t know (pass to level 2)

1. If there are any other reasons to exclude this report, please describe them here (e.g. institutional guideline [e.g. for ethics submission], guideline intended solely for the reporting of non-randomized, non-controlled clinical trials, guideline focused on reporting protocols of a narrow area of health care research, such as a specific medical procedure, condition or laboratory test, tools or checklists intended to assess quality of clinical trial protocols):

______________________________________________________

______________________________________________________

**Screening: Level 2**

**Reviewers Initials: _______________**

**REFID: _______________**

1. Does this paper describe a reporting guideline for protocols?

[ ] No (exclude)

[ ] Yes (include if 2 yes)

[ ] Don’t know (need to resolve)

1. If question above yes: Does this paper describe a reporting guideline for randomized controlled clinical trial protocols? *Note: The guideline may be relevant to other study designs as well.*

[ ] No (exclude)

[ ] Yes (include) **– please** **answer either ‘NO’ or appropriate text to #3**

[ ] Don’t know (need to resolve)

1. If there are any reasons to exclude this report, please describe it here (e.g. institutional guideline [e.g. for ethics submission], guideline intended solely for the reporting of non-randomized, non-controlled clinical trials, guideline focused on reporting protocols of a narrow area of health care research, such as a specific medical procedure, condition or laboratory test, tools or checklists intended to assess quality of clinical trial protocols):

______________________________________________________

______________________________________________________

**Appendix B - Preliminary Data Extraction Form**

Please note that all data will be entered into an SPSS database.

**Identification**

**Reviewers Initials: _______________**

**REFID: _______________**

**Authors: ________________________________________________**

**Journal/Agency: ________________________________________________**

**Country of corresponding**

**author : ______________________________**

**Scope of guideline**

1. Is this reporting guideline exclusively for reporting protocols of randomized controlled trials?

[ ] No

[ ] Yes **– Skip to question 3**

[ ] Unclear/Not reported **– Skip to question 3**

1. If not, what are the other types of study protocols for which this guideline is intended?

_____________________________

_____________________________

_____________________________

[ ] Others not clear

1. Is the reporting guideline intended to inform the reporting of the complete controlled clinical trial protocol?

[ ] No – guiding one portion of the protocol (e.g. reporting of QoL);

[ ] No – guiding one element of the protocol (e.g. ethical considerations); details

of element present in various sections of protocol.

[ ] Yes

[ ] Unclear

**Characteristics of guideline**

1. In what country were these guidelines developed?

___________________

[ ] Don’t know

[ ] Various

1. How is the guideline summarized?

[ ] Text

[ ] Checklist

[ ] Flowchart

**Accountability**

1. Are any authors credited with the development of these reporting guidelines?

[ ] No **– Skip to question 8**

[ ] Yes

[ ] Unclear **– Skip to question 8**

1. If yes to 3, how many? ___________________
2. Is there a date or version number associated with the publication of the reporting guidelines (e.g. date of publication of paper or report)?

[ ] No

[ ] Yes

[ ] Unclear/Not reported

1. Is a contact address provided for authors or departments responsible for the development of the reporting guidelines?

[ ] No

[ ] Yes

[ ] Unclear

**Guideline development process**

1. Are any methods described for the development of the reporting guideline?

[ ] No **– skip to Question 18**

[ ] Yes

[ ] Unclear

1. If so, what methods were described (check all that apply)?

[ ] Systematic literature search

[ ] General (non-systematic) literature search – no explicit methods described

(including elsewhere), no inclusion/exclusion criteria

[ ] Consensus Meeting

[ ] Delphi process

[ ] Informal consensus

[ ] Other (please specify): ___________________

1. How many people were involved in the guideline development process (i.e. by involved we mean have been involved in the development of the guideline or parts thereof)?

_____________ **- if Response = 1, skip to Question 17**

[ ] Not reported - **Skip to Question 17**

1. If greater than 1 person was involved in the guideline development, from which countries were they?

_______________ _______________

_______________ _______________

_______________ _______________

Total # of countries: _______________

[ ] Unclear/Not reported

1. If greater than 1 person was involved in the guideline development, from which disciplines were members of the group?

_______________ _______________

_______________ _______________

_______________ _______________

_______________ _______________

[ ] Unclear/not reported

1. Was a core writing group established?

[ ] No **– Skip to Question 17**

[ ] Yes

[ ] Unclear **– Skip to Question 17**

1. If so, how many people were involved? _______________
2. How long was the time from the inception of guideline development to final guideline (e.g. publication, dissemination, etc.)

______________

[ ] Not reported

**Internal and external validity**

1. Does it appear that evidence was used to inform the development of this reporting guideline.

[ ] No/None described – **Skip to question 20**

[ ] Yes, some items

[ ] Yes, most or all items

[ ] Unclear

1. If so, how was the relevant evidence identified?

[ ] Personal files

[ ] Electronic searches of main databases

[ ] Asking group members, if relevant

[ ] Hand searches of published literature

[ ] Searching ongoing studies

[ ] Searching unpublished literature

[ ] Unclear/Not reported

[ ] Not relevant

1. Was the draft guideline shared with a broader circle of experts for comments before being finalized?

[ ] No

[ ] Shared with working group

[ ] Shared with broader community outside of working group

[ ] Unclear

1. Has this tool been validated?

[ ] No – **Skip to question 23**

[ ] Yes

[ ] Unclear

1. If this tool has been validated, please describe methods of validation.

_______________________________________________________________

_______________________________________________________________

**Dissemination, uptake and impact**

1. How has this guideline been disseminated?

[ ] Publication in one peer-reviewed journal

[ ] Publication in >1 peer-reviewed journal

[ ] Conference presentation

[ ] Website created for guideline

[ ] Other website

[ ] Lectures/talks for potential users

[ ] Unclear/Not reported

1. Has the guideline been formally endorsed by any organizations (by endorsed, we mean that an organization explicitly asked its members to adhere to these guidelines)?

[ ] No

[ ] Yes

[ ] Unclear – to be verified with author, where possible

**Funding**

1. Was any funding received for the development of this protocol reporting guideline?

[ ] No – **Skip to question 28**

[ ] Yes

[ ] Not reported

[ ] Unclear

1. If development of this protocol reporting guideline was funded, please describe source of funding.

[ ] Non-profit – charity

[ ] Non-profit – government

[ ] For-profit – pharmaceutical

[ ] Mixed

[ ] Not reported

[ ] Unclear

1. If development of this protocol reporting guideline was funded, please state which of the following steps was funded (check all that apply).

[ ] Meeting costs

[ ] Travel costs

[ ] Administrative support

[ ] Research

**Items**

1. How many items were included in this reporting guideline? ______

| 29. What items were included in the protocol reporting guideline? (please list each item separately) | Was ‘evidence’ provided for the inclusion of the item?  (Y/N) | If ‘evidence’ was provided, please list types of evidence (e.g. empirical evidence, expert consensus, previous guidelines) with references, where appropriate. |
| --- | --- | --- |
|  |  |  |
|  |  |  |
|  |  |  |
|  |  |  |
|  |  |  |

**Appendix B: Search strategy for Ovid MEDLINE® (including in-process & other non-indexed citations) 1948 to September Week 4 2010**

1. Research design/

2. Clinical protocols/st, mt

3. Clinical Trial as topic/st, mt

4. Biomedical research/

5. Writing/

6. Publishing/st or Evidence-Based Practice/st or Peer review, research/mt, st or Documentation/mt, st

7. or/1-6

8. Protocol$ or Proposal$.ti,sh.

9. (Protocol$ or Proposal$).ab. and (report$ or guideline$ or checklist$ or recommend$ or standard$ or require$ or instruct$ or guidance$ or consensus or Aide memoir$ or writ$).tw.

10. 7 and (8 or 9)

11. clinical trial.pt.

12. animal/ not human/

13. 10 not (11 or 12)

**Appendix C:** Results of contact with nominated trial funding agencies

| **Country** | **Institution/Company** | **Outcome** |
| --- | --- | --- |
| US | National Institutes of Health | Reply received, guidelines included[61,74,96] |
|  | Centers for Disease Control and Prevention | Reply received, guideline included[71] |
|  | Gates Foundation | Reply received, no guideline |
|  | Merck | No reply but guideline included[75] |
|  | Pfizer | No reply but guideline included[76] |
| Japan | Ministry of Health, Labour and Welfare | No reply received, no guideline |
|  | Ministry of Education, Culture, Sports, Science and Technology | No email contact found |
|  | Kidney Foundation | Reply received, no guideline |
|  | Japan Heart Foundation | No reply, no guideline found |
| France | Programme Hospitalier de recherche clinique, French Ministry of health | Reply received, no guideline |
|  | Agence nationale de recherches sur le sida et les hépatites virales | Reply received stating no guideline; draft found but not included due to correspondence. |
|  | Association Francaise contre la myopathie | No email contact found |
|  | Association de recherche contre le cancer | No email contact found |
|  | Servier | No reply, no guideline |
|  | Sanofi | No reply, no guideline |
| Canada | Canadian Institutes of Health Research | Reply received, guideline included[77] |
|  | Alberta Heritage Foundation for Medical Research | Reply received, no guideline |
|  | Heart and Stroke Foundation | Reply received, no guideline |
|  | Hospital for Sick Kids Foundation | Reply received, no guideline |
|  | GlaxoSmith-Kline | No reply, no guideline |
|  | Pfizer (see above) | |
| UK | Medical research council | Reply received, guideline included[46] |
|  | National Institute for Health Research Health Technology Assessment programme | Reply received, no guideline |
|  | Wellcome-Trust | Reply received, guideline included[82] |
|  | Cancer Research UK | Reply received, no guideline |
|  | Glaxo Wellcome | See above (GlaxoSmith-Kline) |
|  | Astra Zeneca | No reply, no guideline found |
| Germany | Federal Ministry of Research and Education/German Research Foundation | Reply received, guideline included[83] |
|  | Deutsche Krebshilfe (German Cancer Aid) | Reply received, guideline not included due to language |
|  | Aesculap AG | No reply, no guideline |

**Appendix D:** Details of guideline development methods described in reports (N = 40 guidelines)

| **Guideline** | **Authors/named contributors*** | **Methods** | **# items/ # concepts** |
| --- | --- | --- | --- |
| NHS Department of Health [UK]: Clinical trials toolkit [46] (2011) | 63 contributors listed; countries unclear | - No methods described - Institutional guide – minimum of informal consensus inferred | 21 / 22 |
| Bridge et al. (2009) - U.S. NIAID Templates. [61] | 14 contributors listed; 1 country | - Informal consensus process and consensus meeting(s); No formal consensus process described - Describes a non-systematic search for previous guidance (personal files) - No search for empirical evidence described - Formal validation plans described and stated to be underway | 109 / 150 |
| Mann, H. (2007) ASSERT statement [73] (RCT-specific) | 1 contributor listed; Ethics board member/ethicist | - No consensus methods described - Empirical evidence cited with guidance for some items - no methods of identifying it described | 18 / 48 |
| Treweek et al. (2006) PRACTIHC tool [48] (RCT-specific) | 15 contributors listed; 11 countries; Methodologists, Statisticians, Other (Trial management, Information Technology, Clinical Application) | - Informal consensus process and consensus meeting(s); No formal consensus process described - Report describes a non-systematic search for previous guidelines (Internet search) and for empirical evidence (Internet search, reference lists, experts) - Field testing and evaluation (used twice as core component in a training course with evaluations received) - Shared with working group, shared with broader community, shared with general public | 40 (in tool) / 42 |
| Malhotra et al. (2005) ROSE [58] (RCT-specific) | 3 contributors listed; 1 country | - No consensus methods described - No search for empirical evidence described - Formal validation - assessed for intra and inter-rater reliability on 8 protocols by 3 different type of assessors (self, fellow student and 2 teachers) on three different occasions - Requests feedback from readers | 10 / 29 |
| International Ethical Guidelines for Biomedical Research Involving Human Subjects. CIOMS / WHO (2002) [44] | 88 contributors listed; 21 countries | - Informal consensus process and consensus meeting(s) - Various face-to-face meetings and electronic correspondence; No formal consensus process described; - Unclear if search conducted for empirical evidence - Guideline was posted and formal comments received from many national and international organizations as well as individuals | 48 / 102 |
| ICH E6. (1998) [72] | Contributors not listed | - Informal consensus process and consensus meeting(s); No formal consensus process described - No search for empirical evidence described - Shared with broader circle of experts outside working group | 54 / 111 |
| Pocock (1983) [60] | 1 contributor listed | - No methods described - Minimal empirical evidence cited - no methods of identifying it described | 14 / 16 |
| Staquet et al. (1980) - E.R.T.C. clinical trial protocols. [55] | 3 contributors listed; 2 countries | - No consensus methods described - Institutional guide – minimum of informal consensus inferred - Empirical evidence cited with guidance for some items - no methods of identifying it described | 18 / 24 |
| Warren (1978) [56] | 1 contributor listed | - No consensus methods described; - No search for evidence described; - Shared with external experts for review of drafts | 13 / 18 |
| Chaput de Saintonge (1977) [57] | 1 contributor listed - ‘produced by past and present members of Clinical Trials Unit, Department of Pharmacology and Therapeutics, London Hospital Medical College’ | - No methods described - Requests feedback from readers on content as well as practical use of list | 86 / 106 |
| Working group of the Commission on Dental Materials, Instruments, Equipment and Therapeutics (1977) [42] | 11 contributors listed; 7 countries | - No methods described | 21 / 24 |
| Schneiderman (1961) [68] | 3 contributors listed; 1 country; | - No methods described - Institutional guide – minimum of informal consensus inferred | 38 / 56 |
| [50,65,71,74-76,82-84,86] | Contributors not listed | - No methods described - Institutional guides – minimum of informal consensus inferred | - |
| [40,52-54,59,62-64,66,67,69,70,78-81,97] | < 3 contributors; 1 country | - No methods described | - |
